# Autosomal recessive retinitis pigmentosa with homozygous rhodopsin mutation E150K and non-coding *cis*-regulatory variants in CRX-binding regions of *SAMD7*

**DOI:** 10.1038/srep21307

**Published:** 2016-02-18

**Authors:** Kristof Van Schil, Marcus Karlstetter, Alexander Aslanidis, Katharina Dannhausen, Maleeha Azam, Raheel Qamar, Bart P. Leroy, Fanny Depasse, Thomas Langmann, Elfride De Baere

**Affiliations:** 1 Center for Medical Genetics, Ghent University and Ghent University Hospital, Ghent, Belgium; 2 Laboratory for Experimental Immunology of the Eye, Department of Ophthalmology, University of Cologne, Cologne, Germany; 3 Department of Biosciences, COMSATS Institute of Information Technology, Islamabad, Pakistan; 4 Al-Nafees Medical College & Hospital, Isra University, Islamabad, Pakistan; 5 Pakistan Academy of Sciences, Islamabad, Pakistan; 6 Department of Ophthalmology, Ghent University Hospital, Ghent, Belgium; 7 Division of Ophthalmology, The Children’s Hospital of Philadelphia, Philadelphia, USA; 8 Department of Ophthalmology, Brugmann University Hospital, Brussels, Belgium

## Abstract

The aim of this study was to unravel the molecular pathogenesis of an unusual retinitis pigmentosa (RP) phenotype observed in a Turkish consanguineous family. Homozygosity mapping revealed two candidate genes, *SAMD7* and *RHO*. A homozygous *RHO* mutation c.448G > A, p.E150K was found in two affected siblings, while no coding *SAMD7* mutations were identified. Interestingly, four non-coding homozygous variants were found in two *SAMD7* genomic regions relevant for binding of the retinal transcription factor CRX (CRX-bound regions, CBRs) in these affected siblings. Three variants are located in a promoter CBR termed CBR1, while the fourth is located more downstream in CBR2. Transcriptional activity of these variants was assessed by luciferase assays and electroporation of mouse retinal explants with reporter constructs of wild-type and variant *SAMD7* CBRs. The combined CBR2/CBR1 variant construct showed significantly decreased *SAMD7* reporter activity compared to the wild-type sequence, suggesting a *cis*-regulatory effect on *SAMD7* expression. As Samd7 is a recently identified Crx-regulated transcriptional repressor in retina, we hypothesize that these *SAMD7* variants might contribute to the retinal phenotype observed here, characterized by unusual, recognizable pigment deposits, differing from the classic spicular intraretinal pigmentation observed in other individuals homozygous for p.E150K, and typically associated with RP in general.

Inherited retinal dystrophies (RDs) affect millions of individuals worldwide. Retinitis pigmentosa (RP, MIM[268000]) is the most prevalent form of RD, with a prevalence of 1 in 4000 and accounting for approximately half of the RD cases. It is a progressive disease that typically presents with degeneration of the rod photoreceptors, followed by loss of cone photoreceptor function. Most patients experience night blindness as an initial symptom. Subsequently, a gradual constriction of the peripheral visual field occurs, often followed by loss of central vision. However, the clinical presentation of RP is highly variable, which is also reflected in an impressive genetic heterogeneity. Inheritance can be autosomal dominant (30–40% of cases), autosomal recessive (50–60%) or X-linked (5–15%)^1^,^2^. More than 70 disease causing genes have been associated with the molecular pathogenesis of RP today, most of which account for only a small percentage of cases^3^. One exception is the rhodopsin (*RHO*) gene, accounting for 30–40% of autosomal dominant RP (adRP) in the North American population^3^,^4^. In contrast to the numerous mutations with an autosomal dominant (AD) inheritance described in this gene, only a few recessive mutations have been reported so far^5^,^6^,^7^,^8^,^9^,^10^. Rhodopsin, which is a photosensitive molecule located in the membranes of the outer segments of rod photoreceptors, consists of a rod-specific opsin bound to the chromophore 11-*cis*-retinal. After absorption of a photon, this chromophore undergoes a conformational change, triggering the phototransduction cascade, converting light into electrical signals^11^.

Despite the large number of RP disease genes that already have been identified, still 40–50% of patients remain without molecular genetic diagnosis^1^. Some of these patients are assumed to have a mutation in a novel, yet to be identified disease gene. The current emergence of whole exome/genome sequencing (WES/WGS) studies in RDs greatly facilitates in identifying these genes^12^,^13^,^14^,^15^,^16^. However, analysis of WES and WGS data is still challenging and additional filtering strategies are needed. One strategy that has been shown to be effective is the use of a Crx-based *cis*-regulatory dataset^13^,^17^,^18^. This dataset consists of a genome-wide map of binding regions of the key retinal transcription factor Crx thereby identifying genes that are specifically regulated by this transcription factor^17^. One of these genes is *Samd7* (sterile alpha motif domain containing 7), which is highly expressed in the adult murine retina and localizes to the outer nuclear layer of the retina, containing the photoreceptor nuclei^17^,^19^. It has been suggested that this gene encodes a transcriptional repressor, involved in fine-tuning of Crx-regulated gene expression^19^. Although this is an excellent candidate RD disease gene, no *SAMD7* mutations have been reported in humans so far.

Besides patients with coding mutations in novel disease genes, other patients remaining without molecular genetic diagnosis could have a yet unidentified mutation in already known RD disease genes, albeit located in functional elements outside the protein coding part of the gene. Examples are mutations in the 5′ untranslated region (UTR), the core promoter of the gene, the 3′ UTR containing microRNA binding sites, deep intronic regions influencing splicing or in more distant regulatory elements such as enhancers^20^,^21^,^22^,^23^,^24^. Several examples of non-coding mutations in such elements have been reported in RD, including a deletion of the first non-coding exon of *EYS*^25^, *LCA5*^26^ and *PCDH15*^27^ containing the promoter of the gene; a non-coding mutation in the first non-coding exon of *EYS*^25^; mutations in the 5′ untranslated region of *NMNAT1*^28^, deep intronic variants in the *ABCA4*^29^,^30^,^31^, *OFD1*^32^, *CEP290*^33^, *USH2A*^34^ and *PROM1*^35^ genes leading to alternate splicing; and a mutation in the seed region of microRNA-204^36^, and very recently variations in a DNase I hypersensitivity site upstream of *PRDM13*^37^.

Here, we report on the molecular pathogenesis in a Turkish consanguineous autosomal recessive RP (arRP) family with unusual nummular retinal pigmentation. The *RHO* mutation E150K was found in a homozygous state in the affected individuals as well as homozygous non-coding SNPs of *SAMD7*. Specifically, these variants were found in non-coding CRX-bound regions (CBRs) associated with the *SAMD7* gene. Luciferase experiments and electroporation assays in mouse retinal explants revealed an effect of these variants on *SAMD7* expression, possibly contributing to the phenotype observed in this family.

## Results

### Clinical evaluation

**Figure 1:**
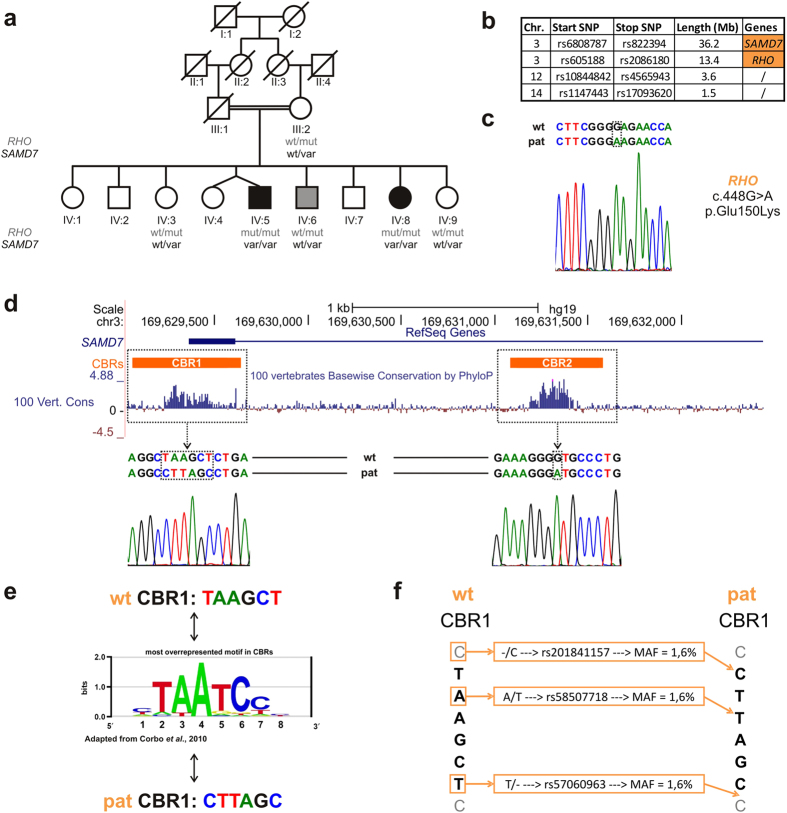
Identification of a homozygous *RHO* mutation and non-coding *SAMD7* variants. (**a)** Pedigree of the family. Six family members participated in this study and were clinically and genetically investigated. The genotype of each individual for the *RHO* mutation is indicated in grey, while the genotype for the *SAMD7* variants is shown in black. The affected siblings IV:5 and IV:8 are homozygous for both variants, while all other family members are heterozygous carriers. One of these siblings IV:6 displays minor subclinical manifestations, more specifically pigmentary anomalies on fundus photography and subjective complaints of night blindness. (**b**) Homozygosity mapping. SNP chip analysis in the affected siblings revealed four shared homozygous regions, harboring the *SAMD7* and the *RHO* genes. Both genes are located on chr. 3, but are not genetically linked as they are 40 Mb apart. (**c**) *RHO* mutation. Electropherogram showing the homozygous *RHO* c.448G > A, p.(Glu150Lys) identified in the affected siblings. Segregation of this mutation is depicted in Fig. 1a. (**d)** *SAMD7*-associated regulatory variants. UCSC genome browser view of the *SAMD7* locus (http://genome.ucsc.edu/). The horizontal blue line in the upper part of the figure represents the structure of the *SAMD7* gene, here only showing the first non-coding exon and a part of the adjacent intron. The orange blocks indicate the *SAMD7*-associated CBRs, with CBR1 located in the promoter region and CBR2 in the first intron. The next track represents the PhyloP conservation scores across 100 vertebrates, showing high conservation of both CBRs. The electropherograms show the homozygous variants located in the CBRs identified in both affected siblings. Segregation analysis of these variants is represented in Fig. 1a. (**e)** Disruption of CRX-binding motif. The middle part depicts the most overrepresented motif in CBRs, as reported by Corbo *et al.*^17^ While the wt CBR1 sequence is very similar to this CRX-binding motif, the variants identified in this study seem to disrupt it completely. (**f**) CBR1 variation. Comparison of the wt and the variant sequences shows that the variation in CBR1 consists of three SNPs with MAF 1.6%: rs201841157, rs58507718 and rs57060963 leading to an insertion, transversion and deletion event, respectively.

Six family members of a Turkish consanguineous family participated in this study: two affected siblings displaying severe RP (IV:5 and IV:8), two healthy siblings (IV:3 and IV:9) and their mother (III:2), and a fifth sibling (IV:6) with a minor subclinical expression of disease (Fig. 1a).

**Figure 2:**
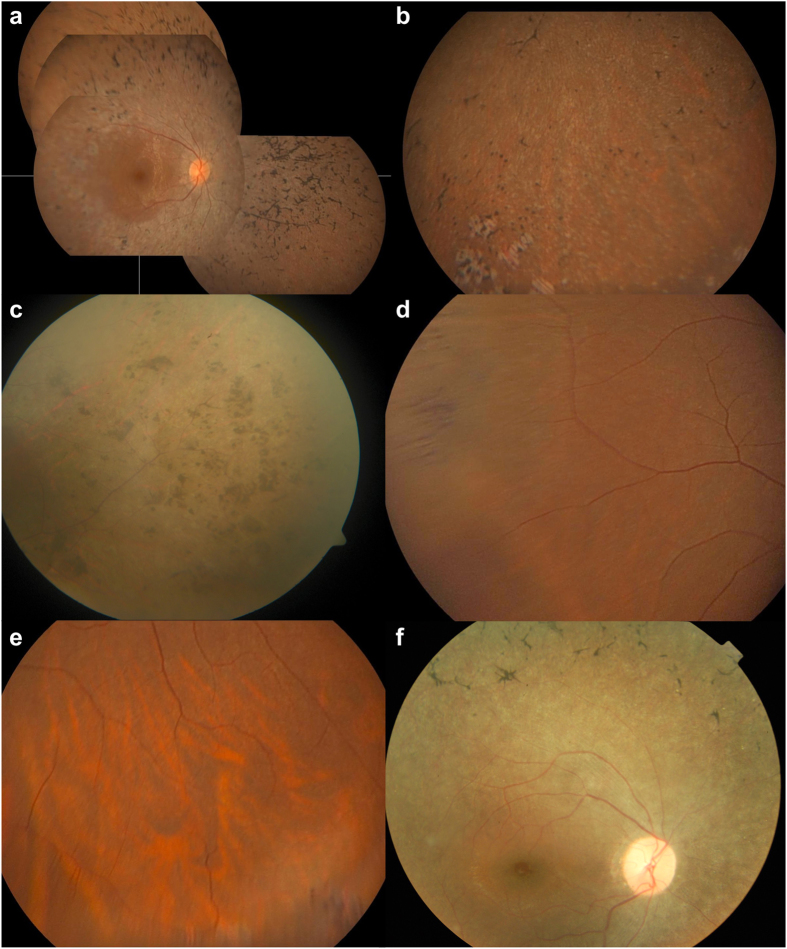
Fundus appearances. (**a,b**) Right eye of affected sibling IV:8, fundus montage images (a), periphery (b). Typical aspect of RP: retinal atrophy with vascular attenuation and relatively preserved macula. Note variation in morphology of pigment deposits: aspect of classic spicular pigmentary changes alternate with unusual nummular types and several well-delineated areas of punched-out retinal atrophy, as seen here in inferior periphery. (**c**) Fundus of left eye of affected sibling IV:5, periphery. Note conglomerates of grouped nummular intraretinal pigmentations in superotemporal periphery in IV:5. (**d,e**). Fundus of right eye of sibling IV:6 with mild subclinical expression of disease, periphery. Multiple zones of peripheral intraretinal pigment migration as mild expression of RP; note difference in aspect of lesions with both spicular and nummular pigmentations combined. (**f**) Fundus of the index patient of Pakistani family RP21^8^. Classic aspect of outer retinal atrophy with spicular intraretinal pigmentation without the unusual and distinct grouped nummular pigmentation seen in the family studied here.

The first affected family member (IV:5), a 49 year-old male, was diagnosed at the age of 20, after previously suffering from night blindness. His best corrected visual acuity (BCVA) is 20/50 in the right eye (RE) and 20/100 in the left eye (LE), with a concentric constriction of Goldmann visual fields to the central 5°. A color vision defect in the blue-yellow axis as well as a subcapsular cataract were observed. On fundus photography, major retinal atrophy was visible, with vascular attenuation and a relatively preserved macula. In the retinal periphery a remarkable aspect of pigmentation was seen. Apart from classic spicular intraretinal pigmentations, conglomerates of grouped nummular pigment deposits were present (Fig. 2c).

The other affected sibling (IV:8), a 44 year-old female, suffers from night blindness and visual field constriction since she was 15 years old. Her BCVA is 20/40 in both eyes, with a concentric constriction of the visual fields to less than 10°. Bilateral posterior subcapsular cataracts were also observed. Fundus imaging showed retinal atrophy, albeit less severe than in the other affected sibling (IV:5). As in her affected brother (IV:5), particular small, nummular intraretinal pigmentations were seen, although not grouped in her. In the periphery, several well-delineated areas of punched-out retinal atrophy were present (Fig. 2a,b).

Extensive ophthalmological examination of two additional siblings (IV:3 and IV:9) and the mother (III:2), was entirely normal.

The phenotype of individual IV:6, a 47-year old male, is particularly interesting. BCVA was measured at 20/30 in the RE and 20/25 in the LE. Visual fields were normal. He claimed to suffer from night blindness, which unfortunately could not be confirmed as the patient did not allow to perform a full field electroretinogram (ERG). Anterior segments were normal. Fundoscopy showed multiple zones of peripheral intraretinal pigmentation with either a spicular or nummular aspect, as well as multiple white dots, representing minor subclinical manifestations (Fig. 2d,e).

### Identification of homozygous *RHO* mutation E150K and non-coding *SAMD7* variants

To identify the underlying genetic cause of RD in this Turkish consanguineous family, homozygosity mapping was performed in the affected siblings IV:5 and IV:8. This revealed several shared homozygous regions, containing the *SAMD7* and *RHO* genes located on two different non-contiguous regions on chromosome 3 (Fig. 1b). Sanger sequencing of the coding regions of *RHO* in one of the affected siblings led to the identification of a rare, yet previously described missense mutation in a homozygous state: c.448G > A, p.(Glu150Lys), also known as E150K (Fig. 1c)^6^,^8^,^10^. Segregation analysis showed that this mutation is homozygously present in the affected siblings IV:5 and IV:8 and that all other family members are heterozygous carriers (Fig. 1a).

In parallel, sequencing of all coding and non-coding exons of *SAMD7* did not reveal any pathogenic changes. However, subsequent analysis of regulatory regions of *SAMD7* in one affected sibling revealed four different variations located in CBRs. Segregation analysis demonstrated that the affected siblings IV:5 and IV:8 are homozygous for these variants, while all other investigated family members are heterozygous carriers (Fig. 1a). *SAMD7* has two previously functionally validated CBRs, one located in the promoter region, CBR1, and the other, CBR2, located in the intronic region between the first two non-coding exons of the gene^17^,^19^. The homozygous variants are known single nucleotide polymorphisms (SNPs), having a minor allele frequency of 1.6% according to dbSNP (http://www.ncbi.nlm.nih.gov/projects/SNP/). Three variants (rs201841157, rs58507718 and rs57060963) are located in CBR1 and consist of an insertion, transversion and deletion event, possibly disrupting a CRX-binding sequence, while the last variant (rs73040228) was found in CBR2 (Fig. 1d–f).

For comparison, family members of two previously described Pakistani arRP families segregating the same E150K *RHO* mutation^8^ were investigated for the presence of the non-coding *SAMD7* variants identified in this study. However, not surprisingly, none of these *SAMD7* variants were detected in both families. In addition, we performed haplotype analysis of the *RHO* region using the flanking markers described by Azam *et al.* This revealed no differences between the disease haplotype here and the previously reported disease-associated haplotype of these two Pakistani families (Supplementary Fig. 1)^6^,^8^.

### SAMD7 localization in human retina

**Figure 3:**
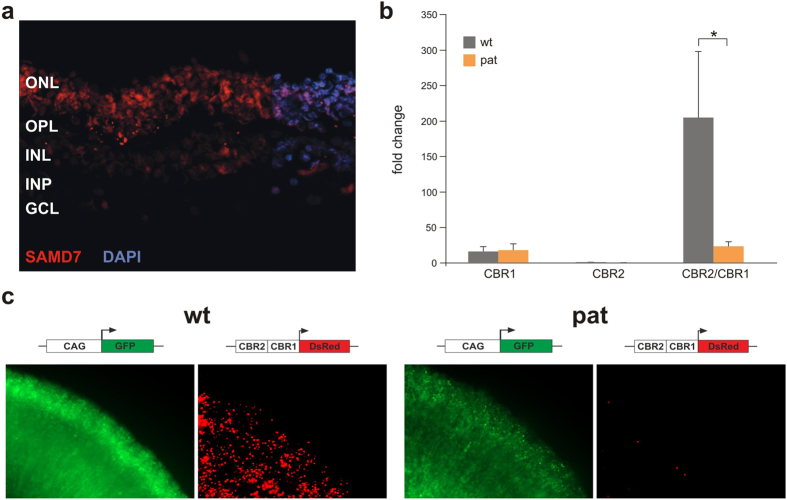
Retinal immunostaining of SAMD7 and transcriptional activity of *SAMD7* variants. (**a**) Human retinal SAMD7 localization. Representative fluorescent images of horizontal cross-sections of human retina stained with anti-SAMD7 antibody (red, 1:250). Retinal counterstaining was performed with 4′,6-diamidino-2-phenylindole (DAPI) (blue). SAMD7 immunoreactivity is predominantly detected in the photoreceptor nuclei, located in the ONL. ONL, outer nuclear layer; OPL, outer plexiform layer; INL, inner nuclear layer; IPL, inner plexiform layer; GCL, ganglion cell layer. (**b**) Luciferase experiments. Luciferase assays were performed in HEK cells, using different *SAMD7* reporter constructs. The first construct consisted of CBR1 cloned in the pGL4.10 reporter vector containing a luciferase expressing gene without promoter. For the second construct CBR2 was cloned in the pGL3 promoter vector, upstream of a luciferase reporter gene and a minimal basal promoter. Finally, the third construct was obtained by cloning CBR2 upstream of CBR1 in the pGL4.10 vector. Two types of each construct were created, one containing the wt sequence of a healthy control, the other one consisting of the patient (pat) sequences containing the *SAMD7* CBR variants. *Cis*-regulatory activity could be demonstrated for the CBR1 constructs, while for CBR2 only very little luciferase expression could be measured. In both cases, no significant difference in luciferase expression has been observed between patient and control. However, when combining CBR2 and CBR1, luciferase expression increases for the control construct, while for the patient construct there is a significant decrease in expression. *corresponds with p < 0.05 after a two-sample t-test. (**c)** Electroporation reporter assays in mouse retinal explants. As a confirmation for the luciferase experiments, electroporation assays were carried out for a CBR2/CBR1 construct in mouse retinal explants. Control and variant CBR2/CBR1 were cloned in a dsRed expressing vector without basal promoter and electroporated into isolated retinas of P0 mice. After eight days of *in vitro* culture, retinas were harvested, fixed and imaged, confirming that the combined *cis*-regulatory activity of CBR2/CBR1 is lost for the patient constructs.

Previously, Samd7 expression has been demonstrated in cross sections of the adult murine retina, showing predominant expression in the photoreceptor nuclei^19^. To investigate the retinal localization of the human SAMD7 protein, immunohistochemical analyses of human *post mortem* retinas were performed. This showed a similar expression pattern as in murine retina, with a distinctive staining pattern in the photoreceptor nuclei (Fig. 3a).

### Assessment of the transcriptional activity of the *SAMD7* variants

*Cis*-regulatory activity of the two murine wild-type (wt) CBRs associated with *Samd7* has been demonstrated previously by electroporation assays in mouse retinal explants^17^,^19^. Here, in order to gain insights into a regulatory effect on *SAMD7* transcription of the SNPs found in CBR1 and CBR2, we wanted to study the *cis*-regulatory effect of the human *SAMD7* CBRs, both for wt CBRs and for variant CBRs of the patients.

First, both wt and variant human CBR1 and CBR2 were cloned in a luciferase expression vector to assess the activity of the CBRs by luciferase assays. CBR1 is located in the proximal promoter region of *SAMD7* and was therefore cloned immediately upstream of the luciferase reporter gene. As CBR2 is located in a more distant enhancer region outside the promoter, it was cloned upstream of a luciferase reporter gene carrying a minimal basal promoter. A third construct consisted of CBR2 cloned upstream of CBR1, in order to assay the combined regulatory activity of CBR2 upstream of CBR1. As depicted in Fig. 3b, no significant difference in luciferase expression could be demonstrated between patient and wt constructs for CBR1 and CBR2 separately. However, variant constructs for which CBR2 was cloned upstream of CBR1 showed a significant 8.8 times decrease in luciferase activity compared to the corresponding wt constructs [wt mean = 204.77, variant mean = 23.31, p = 0.0281] (Fig. 3b).

To confirm these results, electroporation assays in mouse retinal explants have been performed with vectors containing the combined CBRs. CBR1 was cloned upstream of a dsRed reporter gene, followed by upstream cloning of CBR2. Electroporation of this construct in mouse retinal explants substantiated the luciferase data. Indeed, the combined CBR2/CBR1 variant construct again showed a markedly decreased *SAMD7* reporter activity compared with the wild type CBR2/CBR1 construct (Fig. 3c).

## Discussion

The aim of this study was to unravel the molecular pathogenesis of an atypical retinitis pigmentosa phenotype (RP) observed in a Turkish consanguineous family. Homozygosity mapping followed by candidate gene analysis revealed a rare missense mutation in the *RHO* gene, c.448G > A p.(Glu150Lys), also known as E150K, and four non-coding variants in retina-specific regulatory regions of the *SAMD7* gene.

The E150K *RHO* mutation was described for the first time by Kumaramanickavel *et al.* in a large Indian consanguineous family. The four living affected siblings were homozygous for this mutation and displayed typical RP. Two other siblings were heterozygous carriers and displayed no symptoms^6^. Azam *et al.* reported two additional Pakistani arRP families segregating the same *RHO* mutation in homozygous state in affected family members and in heterozygous state in asymptomatic individuals^8^. Recently, this mutation was also reported in a homozygous state in affected individuals of another Pakistani family with classic RP, with no information about heterozygous carriers^10^. Additionally, this mutation has been extensively studied by Zhang *et al.* in E150K knock-in mice. Homozygous KK mice displayed early-onset retinal degeneration, while heterozygous EK mice showed a delayed-onset milder retinal degeneration. Hence, the authors state that the heterozygous *RHO* E150K-associated retinopathy should rather be classified as slowly progressing adRP instead of pure arRP and encourage all human patients carrying this mutation heterozygously to have a follow-up monitoring of their retinal function^38^. So far, no human symptomatic heterozygous carriers of E150K have been reported in literature however.

Most *RHO* mutations have a dominant mode of inheritance. Only a few recessive mutations have been reported: apart from the E150K mutation, only three other recessive mutations have been described, two of them associated with subtle retinal involvement in heterozygous carriers. Rosenfeld *et al.* identified the first *RHO* mutation with autosomal recessive inheritance, c.745G > T, introducing a premature stop codon, p.(Glu249*), in a RP patient of French-Canadian descent originating from a consanguineous marriage. Interestingly, ERGs performed in both parents and heterozygous siblings showed decreased light sensitivity of rod photoreceptors in heterozygous carriers, although no visual field loss could be detected^5^. The second mutation is located in the consensus sequence of the splice donor site of intron 4 (c.936 + 1G > T) and presumably leads to defective splicing of the gene. This mutation was first reported by Rosenfeld *et al.* heterozygously in a healthy control individual, only presenting a subtle abnormality in rod function based on ERG, suggesting an autosomal recessive (AR) inheritance of the mutation^5^. Subsequently, Macke *et al.* and Jacobson *et al.* reported about an adRP family where all four affected family members are heterozygous for this splice site mutation. Disease manifestation varied greatly with age in this family, as the oldest patient had severely decreased visual acuity and undetectable ERG, while the youngest one had a normal fundus, visual acuity and ERG, only showing rod sensitivity loss. This led to the hypothesis of AD inheritance, with a delayed effect of the mutation^39^,^40^. Later, Rosenfeld *et al.* reported two additional families with a total of 25 heterozygous carriers of whom only one 45-year old has been diagnosed with RP during adolescence. Ophthalmological examinations in 11 of the 24 asymptomatic carriers revealed only subtle rod abnormalities, even in four individuals older than 65 years of age. According to the authors, these findings seem to exclude an AD inheritance pattern (penetrance would only be 4%), leaving the possibility of AR/digenic inheritance due to yet unidentified mutations or the involvement of mutations in a different RD gene^41^. Finally, the mutation was identified for the first time in a homozygous state in a South African family, substantiating a recessive effect of this mutation^7^. Finally, Kartasasmita *et al.* identified a homozygous nonsense mutation, c.482G > A [p.(Trp161*)] in two Indonesian families with arRP. Asymptomatic heterozygous family members were found to have a normal fundus, although a slight delay and decrease of b-wave in scotopic ERG response was seen^9^.

The family we describe here is the fifth family reported to segregate the E150K *RHO* mutation, where the affected individuals IV:5 and IV:8 are homozygous, while all other siblings are heterozygous carriers. Of note, one of these siblings (IV:6) displays minor subclinical manifestations, more specifically pigmentary anomalies on fundus photography and subjective complaints of night blindness. However, no remarkable decrease in visual acuity could be objectivized. These findings are of interest in light of the delayed-onset and milder phenotype observed in heterozygous EK mice and the difficult genotype-phenotype correlations for the other recessively inherited *RHO* mutations described above. Interestingly, this individual is the first heterozygous carrier of the E150K mutation in whom subtle pigment deposits could be detected on fundoscopy, although no fundus anomalies were seen in his two heterozygous, asymptomatic sisters. Unfortunately, all family members were reluctant to undergo an ERG, which can be seen as a drawback of this study. Performing an ERG in the heterozygous mildly symptomatic and asymptomatic individuals would allow us to detect subtle decreases in light sensitivity, as seen for the other recessively inherited *RHO* mutations.

The non-coding variants identified in the *SAMD7* region add another level of complexity to the genotype-phenotype correlation in this family. As for the *RHO* mutation, the affected family members IV:5 and IV:8 are homozygous for the four variants, while all other siblings and their mother are heterozygous carriers. This is the first report of human *SAMD7*-associated non-coding variants, for which an effect on transcriptional regulation has been demonstrated by luciferase experiments and electroporation assays in retinal explants. In addition, we demonstrated, for the first time, immunostaining of SAMD7 in human retina. As a potential transcriptional repressor, involved in fine-tuning of CRX-regulated gene expression^19^, reduced or abolished *SAMD7* expression could alter the expression of other RD genes. Even though the primary genetic defect has been identified in this family, specifically a well studied missense mutation in *RHO*, a modifying effect of the regulatory *SAMD7* variants on the observed phenotype cannot be excluded. Interestingly, in addition to the spicular pattern of pigment typically seen in RP, distinct conglomerates of grouped nummular pigmentations and several well-delineated areas of punched-out atrophy could be observed in the two affected siblings here. Comparison of the fundus photographs of these Turkish patients with those of the index patient of Pakistani family RP21 segregating the E150K *RHO* mutation^8^, but not the *SAMD7* variants, revealed a classic RP pigmentation and absence of the distinct nummular retinal pigmentation in the Pakistani patient. These phenotypic differences between patients with the same primary mutation might be attributed to modifying genetic factors, like the *SAMD7* variants we identified in this study. While the same atypical, recognizable retinal pigmentation was also seen in the extreme periphery in the heterozygous brother, both healthy heterozygous sisters and their mother appear to have completely normal fundus photographs. This means that the subtle fundus abnormalities seen in the heterozygous brother cannot only be explained by the *RHO* and *SAMD7* genotypes, assuming the presence of other modifying factors.

At present, little is known about modifying genes in human RDs. While some examples have been described for mice and dogs^42^,^43^,^44^,^45^,^46^, the first report in humans dealt with a known *AIPL1* mutation p.(Arg302Leu) identified as a potential modifier allele in a patient with Leber congenital amaurosis (LCA) and prominent maculopathy carrying two *CRB1* mutations^47^. Later, a variant in *RPGRIP1L* has been associated with the development of RD in individuals with ciliopathies caused by mutations in other genes^48^; *AHI1* mutations were presented as potential neurological modifiers of *CEP290*-related disease^49^; in addition to previous studies showing that penetrance of *PRPF31* mutations in adRP correlates with the expression level of the remaining wt *PRPF31* allele^50^,^51^, *CNOT3* was proposed as a modifier of *PRPF31* mutations in RP with incomplete penetrance^52^,^53^; and more recently it has been suggested that a specific *NMNAT1* variant could act as a modifier in other genetic subtypes of LCA^54^. In this study we showed a potential *cis*-regulatory role of upstream non-coding SNPs on *SAMD7* expression, which might contribute to the retinal phenotype observed here. Further studies are needed however to provide more evidence for a primary or modifying role of respectively *SAMD7* coding and non-coding variation in RD pathogenesis.

In conclusion, we identified the rare missense mutation E150K in the *RHO* gene in a Turkish consanguineous RD family, together with non-coding variants impairing *cis*-regulatory activity of *SAMD7*-associated CBRs, which might contribute to the phenotype observed in this family, characterized by a specific unusual pigmentation, in addition to classic RP characteristics.

## Methods

### Clinical evaluation

Six family members of a Turkish consanguineous arRP family participated in this study: five siblings (IV:3, IV:5, IV:6, IV:8 and IV:9) and their mother (III:2). All family members underwent an ophthalmological examination, consisting of visual acuity measurement, slit lamp examination and fundoscopy. We completed the examination with Goldmann visual field and optical coherence tomography for the two affected siblings (IV:5 and IV:8) and sibling IV:6. Informed consent was obtained, and research protocols adhered to the tenets of the Declaration of Helsinki and were approved by the ethical committee of Ghent University (PA2011/022).

### Molecular genetic evaluation

#### Identity-by-descent and homozygosity mapping

This was performed in the two affected siblings by genome-wide single-nucleotide polymorphism chip analysis using the HumanCytoSNP-12 BeadChip platform (Illumina, San Diego, CA). Identity-by-descent regions (>1 Mb) were identified using PLINK software^55^ integrated in ViVar^56^. Resulting homozygous regions were ranked according to their length and number of consecutive homozygous single-nucleotide polymorphisms, as described by Coppieters *et al*^57^.

#### Sanger sequencing of candidate genes

Primers for the coding exons of *RHO* (NM_000539.3), both coding and non-coding exons of *SAMD7* (NM_182610.2) and the *SAMD7*-associated CBRs were designed using Primer3Plus (http://www.bioinformatics.nl/cgi-bin/primer3plus/primer3plus.cgi). All primer sequences can be found in Supplementary Table 1. Sanger sequencing was performed on an ABI3730XL genetic analyzer using the BigDye Terminator v3.1 Cycle Sequencing Kit (Applied Biosystems, Foster City, CA). Sequences were analyzed with Seqscape software (Applied Biosystems). All genomic positions are based on genome build GRCh37/hg19.

#### Haplotype analysis

Primers were designed for the three SNPs used in the haplotype analysis described by Azam *et al.* (rs789231, rs2855557 and rs2625961), followed by Sanger sequencing and determination of the genotype for each SNP in all family members of the Turkish family and the described Pakistani family RP21^8^. Primer sequences and SNP details are listed in Supplementary Table 1 and 2.

### SAMD7 immunohistochemistry in human retina

#### Retinal samples of human donors

Retinal samples of human donors were obtained from the Eye Bank of the Center of Ophthalmology, University of Cologne, Germany. The research followed the tenets of the Declaration of Helsinki. After dissection of the anterior segment, the remaining tissue included the posterior pole. Remaining vitreous humor was removed to obtain retinal tissue before further processing.

#### Immunohistochemistry

For immunofluorescence analysis, horizontal human retinal cryo-sections were fixed with 4% paraformaldehyde and rinsed with PBS. Sections were then rehydrated in PBS and preincubated with 1% dried milk in PBS and 0.01% Tween 20 to reduce nonspecific immunoreactivity. Overnight incubation with the primary anti-SAMD7 Q-12 antibody (sc100141, Santa Cruz Biotechnology, Dallas, TX) was performed at 4 °C in PBS containing 2% BSA, 0.02% NaN3 and 0.1% Triton X-100. To estimate the specificity of the primary antibody, control stainings without primary antibody were performed in parallel. After washing in PBS, samples were labeled for 1 h at room temperature with the secondary anti-rabbit antibody conjugated to Alexa594 (red) (Dianova, Hamburg, Germany). Nuclei counter-staining was performed with 0.1 mg/ml DAPI (4′,6-diamidino-2-phenylindole) in PBS (Molecular Probes, Life Technologies, Frankfurt, Germany) for 10 min at room temperature. The cryo-sections were mounted with fluorescent mounting medium (Dako Cytomation, Hamburg, Germany) and viewed with a Zeiss Axio Imager.M2 fluorescence microscope equipped with ApoTome.2 (Carl Zeiss, Jena, Germany). Microscopic pictures were analyzed with ZEN software (Carl Zeiss, Jena, Germany).

### Functional evaluation of *SAMD7* variants

#### Luciferase assays

Human CBR1 and CBR2 were PCR amplified in a healthy control individual and one of the affected siblings, leading to a 594 bp and 694 bp amplicon, respectively. The primer sequences are listed in Supplementary Table 1. Using NheI and BglII restriction enzyme (RE) sites CBR1 was cloned in the pGL4.10 reporter vector (Promega, Madison, WI) containing the luciferase expressing gene *luc2* without promoter. CBR2 was cloned in the pGL3 promoter vector (Promega), upstream of a luciferase reporter gene *luc* + and a SV40 promoter, using the KpnI and NheI REs. Finally, using the same set of RE sites CBR2 was cloned upstream of CBR1 in the pGL4.10 vector. All clones were validated using vector-specific and internal primers. The *SAMD7* reporter vectors were co-transfected with either a *CRX*-expressing vector (pcDNA4 CRX) or a control vector (pcDNA4HisMaxA) in HEK cells. HEK cells do not normally drive photoreactive gene expression and will need Crx co-transfection to drive luciferase expression. Transfection was carried out in 12 well plates using 100 μL of serum-free medium, 0,2 μg of vector DNA and 3 μL of *Trans*IT^®^-LT1 Transfection Reagent (Mirus, Madison, WI) per vector per well. After 48 h, luciferase activity was measured on an Infinite F200 Pro plate reader (Tecan, Crailsheim, Germany), using 20 μL of cell lysate and 100 μL of luciferase assay reagent (Promega) per well. Luciferase activity was measured as fold change compared to empty plasmid. All assays have been performed in three independent experiments, using three replicates in each experiment.

#### Electroporation assays

As a confirmation for the luciferase assays, electroporation assays were carried out with CBR2/CBR1 vectors. CBR1 was cloned in a dsRed expressing vector without basal promoter using XbaI and KpnI RE sites, followed by upstream cloning of CBR2 with SalI and XbaI REs. DNA cocktails containing the *SAMD7* reporter vectors and a pCAG-GFP vector as an electroporation control were electroporated in isolated retinas of P0 mice. After eight days of *in vitro* culture, retinas were harvested, fixed and imaged. The detailed protocol used for the electroporation assays has been described previously^58^.

## Additional Information

**How to cite this article**: Van Schil, K. *et al.* Autosomal recessive retinitis pigmentosa with homozygous rhodopsin mutation E150K and non-coding *cis* -regulatory variants in CRX-binding regions of *SAMD7. Sci. Rep.* **6**, 21307; doi: 10.1038/srep21307 (2016).

## Supplementary Material

Supplementary Information
