# Supplementary material for: Autosomal recessive retinitis pigmentosa with homozygous rhodopsin mutation E150K and non-coding cis-regulatory variants in CRX-binding regions of SAMD7
Source: Sci Rep. 2016 Feb 18;6:21307. doi: 10.1038/srep21307 (PMC4758057; doi:10.1038/srep21307)
Supplement: Supplementary Information [file srep21307-s1.doc]

**SUPPLEMENTARY INFORMATION**

**Autosomal recessive retinitis pigmentosa with homozygous rhodopsin mutation E150K and non-coding *cis*-regulatory variants in CRX-binding regions of *SAMD7***

Kristof Van Schil, MSc^1^, Marcus Karlstetter, PhD^2^, Alexander Aslanidis, MSc^2^, Katharina Dannhausen, MSc^2^, Maleeha Azam, PhD^3^, Raheel Qamar, PhD^3,4,5^, Bart P. Leroy, MD, PhD^1,6,7^, Fanny Depasse, MD^8^, Thomas Langmann, PhD^2^, Elfride De Baere, MD, PhD^1*^

^1^Center for Medical Genetics, Ghent University and Ghent University Hospital, Ghent, Belgium

^2^Laboratory for Experimental Immunology of the Eye, Department of Ophthalmology, University of Cologne, Cologne, Germany

^3^Department of Biosciences, COMSATS Institute of Information Technology, Islamabad, Pakistan

^4^Al-Nafees Medical College & Hospital, Isra University, Islamabad, Pakistan

^5^Pakistan Academy of Sciences, Islamabad, Pakistan

^6^Department of Ophthalmology, Ghent University Hospital, Ghent, Belgium

^7^Division of Ophthalmology, The Children’s Hospital of Philadelphia, Philadelphia, USA

^8^Department of Ophthalmology, Brugmann University Hospital, Brussels, Belgium

^*^Corresponding author:

Elfride De Baere, MD, PhD,

Elfride.DeBaere@UGent.be

Center for Medical Genetics Ghent

Ghent University

De Pintelaan 185

B-9000 Ghent

Belgium

Phone: +32-9-332.5186

Fax: +32-9-332.6549

**Supplementary Table S1. PCR primers.**

| **Primers (5’-3’)** |  |  |
| --- | --- | --- |
| *RHO coding regions* |  |  |
|  | F-primer (5’-3’) | R-primer (5’-3’) |
| *RHO -* exon 1A | AATCTCCCAGATGCTGATTC | AGGTTGAGCAGGATGTAGTT |
| *RHO -* exon 1B | CTGAGCCATGGCAGTTCT | CATTGACAGGACAGGAGAAG |
| *RHO -* exon 2 | GGGAGTGCACCCTCCTTAG | GTCCTGACTGGAGGACCCTA |
| *RHO -* exon 3 | CAGACGTTTATGATCCCCTTTT | TCCAGACCATGGCTCCTC |
| *RHO -* exon 4 | GCATGCATCTGCGGCT | CTGCTGCCAGTAACCCTGAT |
| *RHO -* exon 5 | CACTAACGTGCCAGTTCCAA | TCATTCTGCACAGGCGC |
|  |  |  |
| *RHO haplotype analysis* |  |  |
|  | F-primer (5’-3’) | R-primer (5’-3’) |
| *RHO* - rs789231 | AAGGTTGAGGTCTCCCCAAG | AGACATCTGCTCCCTGTGCT |
| *RHO* - rs2855557 | CTCACTCAGGTGGGAGAAGC | TCTCCGAGGAACCCTTTACC |
| *RHO* - rs2625961 | AACACAGGAAAGTGGCTGCT | GGCCTGCTCCTAGAACACTG |
|  |  |  |
| *SAMD7 coding + non-coding regions* |  |  |
|  | F-primer (5’-3’) | R-primer (5’-3’) |
| *SAMD7* - 5upA - upstream - CBR1 | AGGGTTTTCTCACTGCCTGT | CTTTGCCCTCTGATATCCTCT |
| *SAMD7* - exon 1 - 5’ UTR - CBR1 | CAGGTTTATGCACCTTGCAG | GCATTTCCTTTAAGATAAAGATGTCC |
| *SAMD7* - intron 1A - CBR2 | TCAGAAAACATGCATCATACATACC | CCCAGCACATTAGGAGGATG |
| *SAMD7* - intron 1B - CBR2 | TCATCTATGTGCTGCCTGGA | GGCAACGTTCTAAGGTTGATCT |
| *SAMD7* - exon2 - 5’ UTR | TACTCCTTCACACGGGCTTC | GGAAAAAGGAGAAGGAAAAAGG |
| *SAMD7* - exon 3 | AAAGGCTTTTATTGTGTTCAAGAAG | TCCAAAACGTAATGATTGTCCA |
| *SAMD7* - exon 4 | GCCATGGAAATTGTGTACTCC | ATGCATCCATCCAGTTCAAA |
| *SAMD7* - exon 5 | GCAATGATCTGATACCATTTCCA | TGTTTCTTTTTGTAATCTGCCTGA |
| *SAMD7* - exon 6A | TTTCCACCAAACAAACTTCAGAT | CATAGGGAACTGCATGAGTCTG |
| *SAMD7* - exon 6B | AAGCTCTAGACAGTGATGCTGAGA | CGGACTTGGCAACCTGTAGT |
| *SAMD7* - exon 7 | CAAGACCACTCTGGGGTGATA | TGACCTAATTCATTATGCAGGAAA |
| *SAMD7* - exon 8 | TTTTTGTCATCCTCAAGCAGTC | CTCAACCACAGAGGGAAAGC |
| *SAMD7* - exon 9A | AGATGCTCCATGCCTGAAAT | TCTCCAGTCATTTGGTGCTG |
| *SAMD7* - exon 9B - 3’ UTR | CTTTCTGGGGCTTTCATGG | CTGCTTCACGCTGGAAATCT |
| *SAMD7* - exon 9C - 3’ UTR | GAGAAGCTGACCACGACTCC | CCCTCCTTGAGCAAACTGTC |
| *SAMD7* - 3downA - downstream | TGCTCACACCCTGTGGAAT | TCAGAAGATCCATTTCCAAGTT |
| *SAMD7* - 3downB - downstream | TTTCTCACTCTTGGGGAAGC | CCCACCTTCCAAATCAAAAA |
| *SAMD7* - 3downC - downstream | TGTGTTGTTTTCAGCCACTAAG | GACCAAACAGTTCTTCGGTACA |
| *SAMD7* - 3downD - downstream | AAGCCAATGAATAACGAGTGG | TCTTACAATGAAGAGTACTGAGAGCTG |
| *SAMD7* - 3downE - downstream | CACTGTGCTTAATTTTAGGTCCAT | CAGCTGCATAGTACTCCATTGC |
| *SAMD7* - 3downF - downstream | TCTTGTTGGAAAACCATCACA | TCCAAAACTTTTACATCTGGGTTA |
| *SAMD7* - 3downG - downstream | AGGGAATGGGAAAAGGAAAA | GCTCATGTGTTCTGCCAGTC |
| *SAMD7* - 3downH - downstream | GGGAAATCTGAACACAATTTGG | CTGGGTGGACCAAGAGACTG |
| *SAMD7* - 3downI - downstream | AGCAAGCCTGCTCTTTGTTC | GCCCAGCCCTATACTCTTTTT |
| *SAMD7* - 3downJ - downstream | TGAAGCTGGGTTTTGACTACA | AACTAATGGAAACAATTTAATGGAA |
| *SAMD7 luciferase and electroporation assays* |  |  |
| *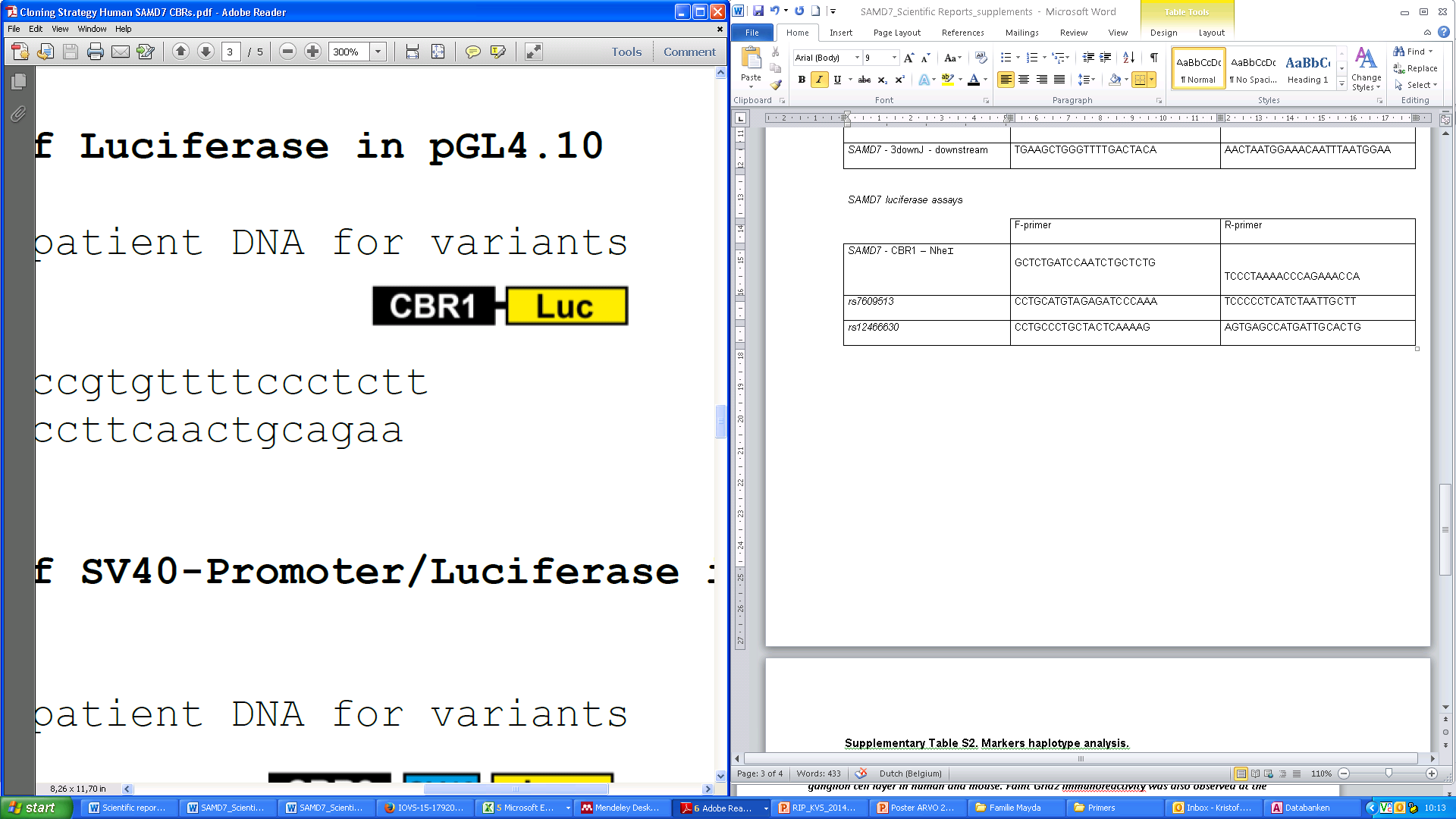* | *SAMD7* - CBR1 - NheI - F-primer | ccc**GCTAGC**aaattccgtgttttccctctt |
|  | *SAMD7* - CBR1 - BglII - R-primer | ccc**AGATCT**ctggccttcaactgcagaa |
| *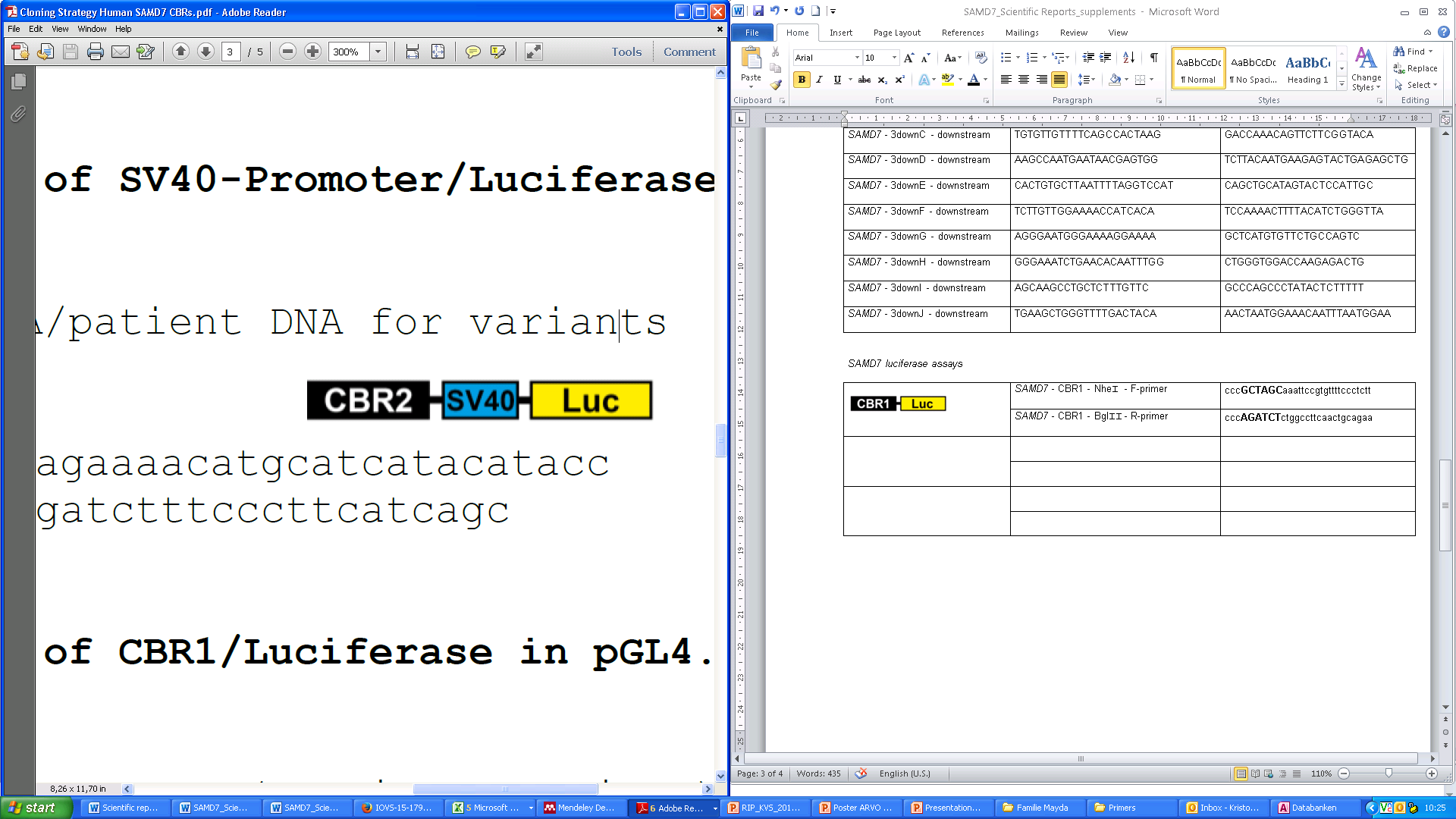* | *SAMD7* - CBR2 - KpnI - F-primer | ccc**GGTACC**tcagaaaacatgcatcatacatacc |
|  | *SAMD7* - CBR2 - NheI - R-primer | ccc**GCTAGC**ctgatctttcccttcatcagc |
| *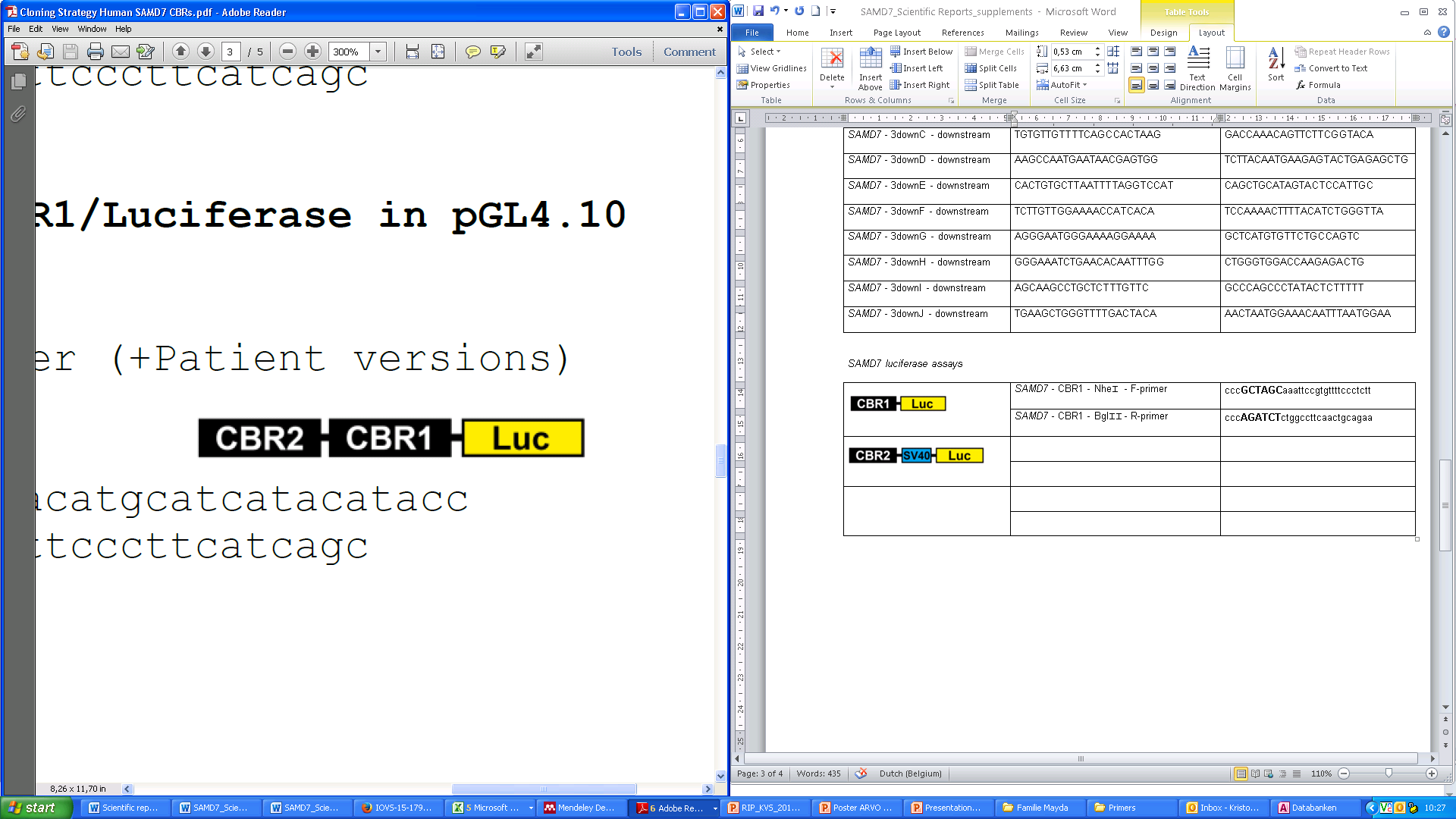* | *SAMD7* - CBR2 - KpnI - F-primer | ccc**GGTACC**tcagaaaacatgcatcatacatacc |
|  | *SAMD7* - CBR2 - NheI - R-primer | ccc**GCTAGC**ctgatctttcccttcatcagc |
| *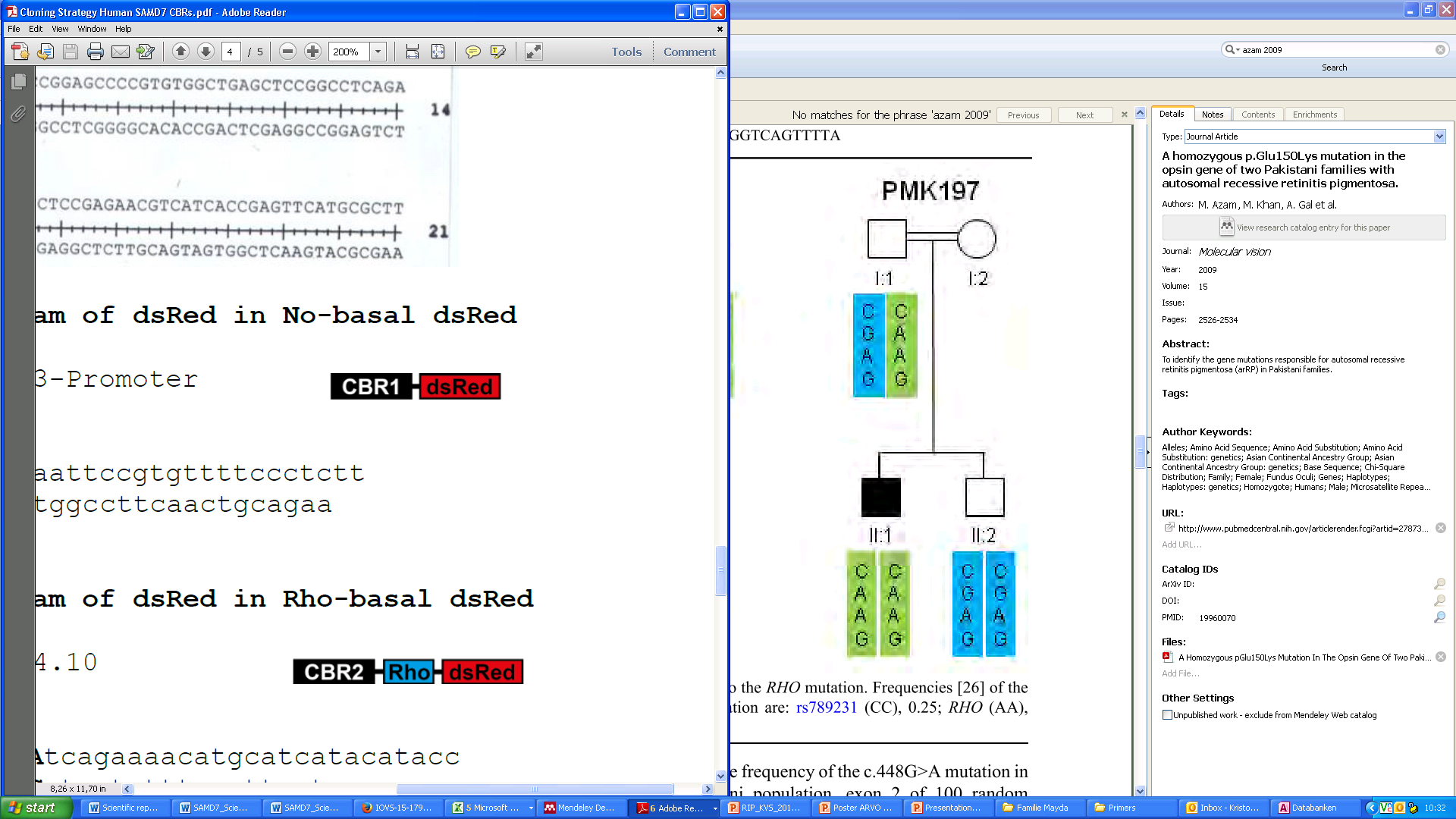* | *SAMD7* - CBR1 - XbaI - F-primer | ccc**TCTAGA**aaattccgtgttttccctctt |
|  | *SAMD7* - CBR1 - KpnI - R-primer | ccc**GGTACC**ctggccttcaactgcagaa |
| *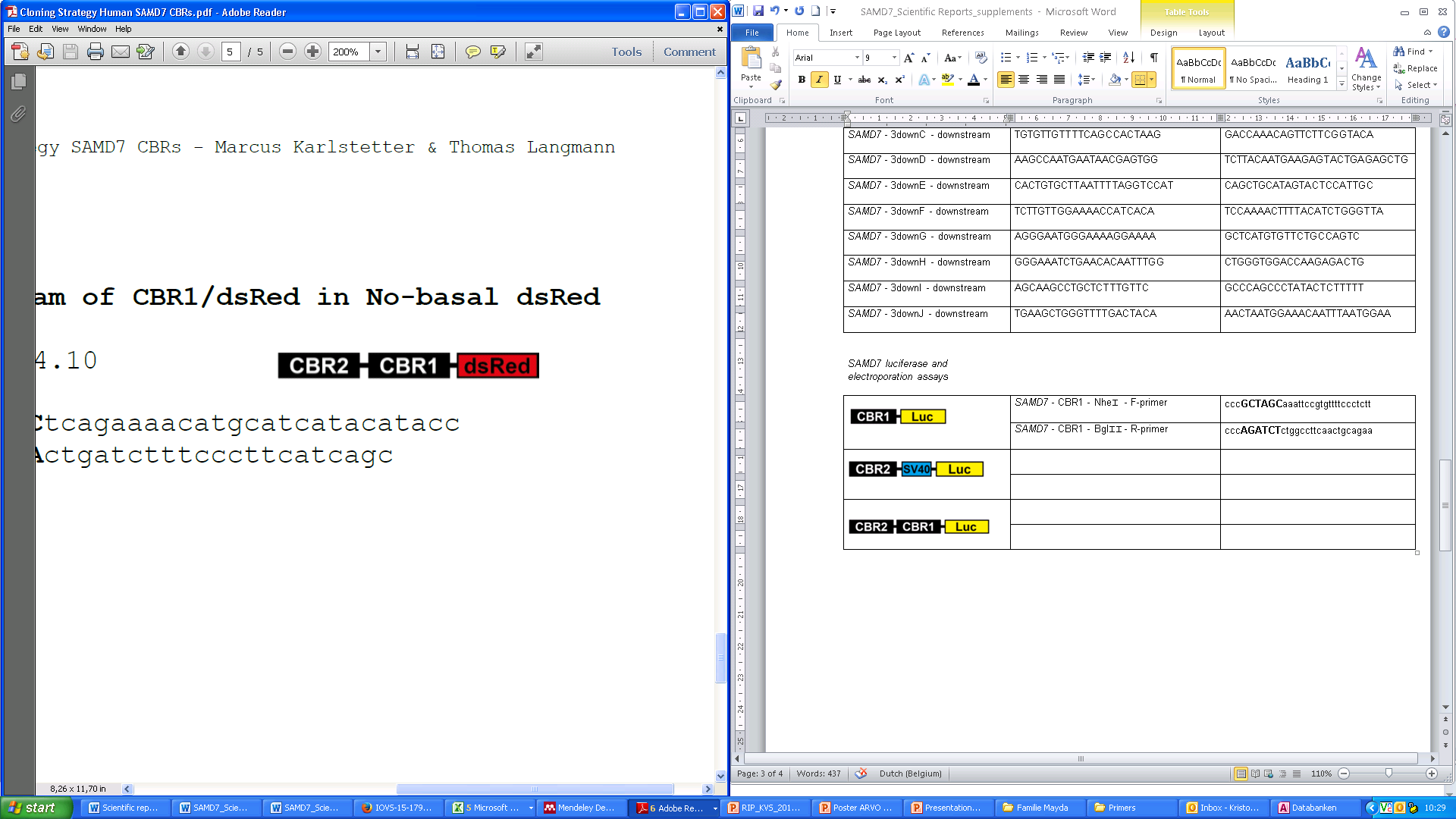* | *SAMD7* - CBR2 - SalI - F-primer | ccc**GTCGAC**tcagaaaacatgcatcatacatacc |
|  | *SAMD7* - CBR2 - XbaI - R-primer | ccc**TCTAGA**ctgatctttcccttcatcagc |

**Supplementary Table S2. Markers haplotype analysis.**

| **SNP** | **Major / minor allele** | **Location relative to *RHO*** | **Gen. position (hg19)** | **MAF (in %)** |
| --- | --- | --- | --- | --- |
| rs789231 | A>G | 5' upstream | chr3: 128,602,847 | 45,2 |
| rs2855557 | T>A | intron 4 | chr2: 129,251,922 | 47,8 |
| rs2625961 | C>T | 3' downstream | chr2: 129,296,020 | 34,2 |
|  |  |  |  |  |

**Supplementary Figure S1. Haplotype analysis.**

**
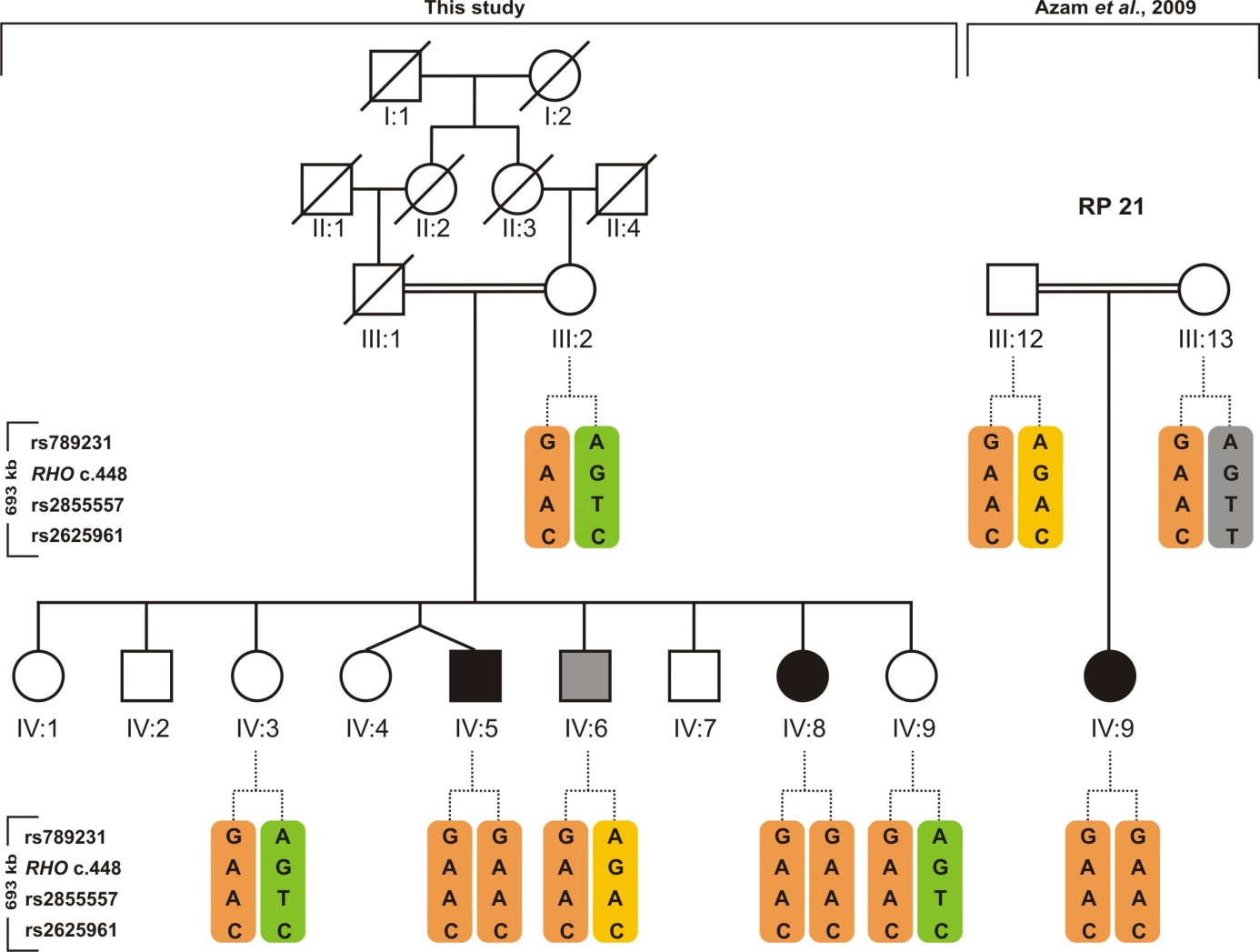
**

Haplotype analysis using three SNP markers spread over a region of 693 kb has been performed in the six available family members of the Turkish family presented in this study. The same markers have been used by Azam *et al*. for the haplotype analysis of the two Pakistani families, of which family RP21 is shown in the right part of the figure. As *RHO* is located on the positive DNA strand, the genotype for each SNP is also based on the positive strand. Based on these three SNPs no difference could be observed between the disease-associated haplotype identified in the Turkish and Pakistani family.
